# Infections and antimicrobial resistance in an adult intensive care unit in a Brazilian hospital and the influence of drug resistance on the thirty-day mortality among patients with bloodstream infections

**DOI:** 10.1590/0037-8682-0106-2019

**Published:** 2020-06-22

**Authors:** Sebastiana Silva Sabino, Caio Augusto de Lima, Luiz Gustavo Machado, Paola Amaral de Campos, Astrídia Marília de Souza Fontes, Paulo Pinto Gontijo-Filho, Rosineide Marques Ribas

**Affiliations:** 1 Universidade Federal de Uberlândia, Faculdade de Medicina, Uberlândia, MG, Brasil.; 2 Universidade Federal de Uberlândia, Instituto de Ciências Biomédicas, Uberlândia, MG, Brasil.

**Keywords:** Intensive care unit, Infections associated with health care, Multidrug-resistant organism, Bloodstream infections

## Abstract

**INTRODUCTION:**

The present study aimed to determine the incidence of health care-associated infections (HCAIs) and identify the main resistant microorganisms in intensive care unit (ICU) patients in a Brazilian university hospital.

**METHODS:**

A retrospective cohort study was conducted in a Brazilian teaching hospital between 2012 and 2014.

**RESULTS:**

Overall, 81.2% of the infections were acquired in the ICU. The most common resistant pathogenic phenotypes in all-site and bloodstream infections were oxacillin-resistant coagulase-negative staphylococci and carbapenem-resistant *Acinetobacter* spp. (89.9% and 87.4%; 80.6% and 70.0%), respectively.

**CONCLUSIONS:**

There is an urgent need to focus on HCAIs in ICUs in Brazil.

Hospital infections, as well as mortality rates, remain higher in low- and middle-income countries than in developed countries^1^ ^,^ ^2^. Furthermore, the frequency of intensive care unit (ICU)-acquired infections and antimicrobial resistance rates are at least two- to three-fold higher in such countries^3^. Brazilian hospitals face major challenges in detecting and controlling antimicrobial resistance, and the limitations in microbiological assessments are highlighted by the scarce information available on antimicrobial resistance^4^ ^,^ ^5^. Risk factor recognition may be related to this scenario, particularly in ICUs at the regional level. Hence, because risk factors create complications during antibiotic prescription - which requires clinicians to consider multidrug-resistant bacteria - predisposing individuals to poor outcomes, risk recognition is an essential component of preventive strategies^2^ ^,^ ^6^. The increase in antibiotic resistance is a persistent issue. Compared with Europe and the United States, Brazil and other Latin American countries have higher levels of antimicrobial resistance in non-fermentative gram-negative bacilli (GNB) and *Enterobacteriaceae* producing extended-spectrum β-lactamase, as well as in some gram-positive organisms (including *Staphylococcus aureus*)^7^ ^,^ ^8^ ^,^ ^9^.

The present study aimed to determine the incidence of health care-associated infections (HCAIs) and identify the main resistant microorganisms in adult patients admitted to ICUs at a teaching hospital in Brazil.

The study was conducted at a tertiary care teaching hospital affiliated with the Federal University of Uberlândia, Minas Gerais, Brazil. This hospital has 530 beds, of which 30 are in the adult ICU. Incidence data were obtained from consecutive patients admitted to the adult ICU between 2012 and 2014. Bacterial identification and susceptibility tests were performed in the hospital laboratory using a VITEK-2^®^ Automated System (bioMérieux). The study was approved by the Research Ethics Committee under protocol number 1.627.990.

Patients admitted to the unit between January 1, 2012 and December 31, 2014 were considered “new patients.” All patients, both new and old, were counted once during the study and all patients who were re-hospitalized during the study period and who remained in the ICU for less than 24 hours were excluded^10^. HCAIs were defined according to the Center for Disease Control and Prevention (CDC Atlanta, USA) criteria. 

The overall HCAI frequency was estimated by calculating the cumulative incidence (number of cases of HCAIs/total number of patients included in the study). The total mortality rate was also estimated, and different microorganisms isolated from monomicrobial or polymicrobial infections were identified.

Over the 3-year study period, a total of 2168 patients (both new and old) with a mean age of 55 years (37.2% female) were enrolled in the study. Among patients infected with antimicrobial resistant pathogens, factors associated with HCAIs included nephropathy, surgery, trauma, and invasive procedures.

A total of 1979 HCAI episodes were observed in these patients, with an incidence rate of 55.1%. Most nosocomial infections were acquired in the ICU (81.2%), while the remainder (18.8%) were acquired in other wards. Bloodstream (33.4%) and lung (30.5%) infections were the most common, followed by urinary tract infections (16.6%). Overall, 1722 of the 1979 episodes were monomicrobial and 257 were polymicrobial. The mortality rate among patients who developed HCAIs was 37.8% (Table 1).

**TABLE 1:** Description and epidemiological indicators of nosocomial infections in the adult intensive care unit assessed in the present study.

Variables	N/Total (%)
Number of patients included in the study	2168
Number of new patients	1576
Cumulative incidence (CI)	869/1576 (55.1)
HCAI* acquired in the hospital	163/869 (18.7)
HCAI acquired in the intensive care unit	706/869 (81.2)
Total episodes of infection	1979/2168 (91.3)
Blood	661/1979 (33.4)
Lung	605/1979 (30.5)
Urine	329/1979 (16.6)
Surgical site	121/1979 (6.1)
Others**	263/1979 (13.2)
Monomicrobial etiology	1722/1979 (87.0)
Mixed etiology	257/1979 (13.0)
Total mortality (%)	416/1576 (26.4)
Thirty-day mortality among patients with HCAIs in the ICU	267/706 (37.8)

*HCAIs = Health care-associated infections; **Catheter tip (n=239); Spinal head fluid (n=6); Tissue fragment (n=1); Other secretions (n=17).

The microbiological analysis of the pathogens and the major resistant phenotypes in HCAI infections at all sites and in the bloodstream are presented in Table 2. Overall, of the 2217 isolates obtained, gram-positive cocci (GPC) were identified in 33.7%, GNB in 56.1%, and yeast in 10.1%. Overall, among the GPC, coagulase-negative staphylococci (CoNS) (58.6%, 438/748) were the most common, while among the GNB, *Pseudomonas aeruginosa* (27.4%, 341/1245) was the most common, followed by *Acinetobacter baumannii* (20.3%, 253/1245) and *Klebsiella* spp. (14.5%, 180/1245).

**TABLE 2:** Epidemiologically important isolated microorganisms and antibiotic resistance phenotypes in patients in the adult intensive care **u**nit assessed in the present study (January 2012 to December 2014).

Microorganisms/Phenotypic resistance	Prevalence of microorganisms			
	All sites		Blood	
	N* = 2217 (%)	N* = 1257 (%)	N* = 787 (%)	N* = 494 (%)
*Staphylococcus aureus/*oxacillin	219 (9.8)	124 (56.6)	41 (5.2)	29 (70.7)
Coagulase-negative *Staphylococcus* spp./ oxacillin	438 (19.7)	394 (89.9)	356 (45.2)	311 (87.4)
*Enterococcus* spp./vancomycin	76 (3.4)	10 (13.2)	30 (4.0)	5 (16.7)
Others (gram-positive cocci)	15 (0.7)	-	6 (0.8)	-
Total	748 (33.7)	528 (42.0)	433 (55.0)	345 (79.7)
*Escherichia coli*/3^rd^ and 4^th^ g. cephalosporins	96 (4.3)	42 (43.8)	16 (2.3)	5 (31.2)
*Klebsiella* spp./3^rd^ and 4^th^ g. cephalosporins	180 (8.1)	138 (76.7)	43 (5.5)	28 (65.1)
*Enterobacter* spp./3^rd^ and 4^th^ g. cephalosporins	137 (6.2)	91 (66.4)	44 (5.6)	27 (61.4)
*Serratia* spp./3^rd^ and 4^th^ g. cephalosporins	138 (6.2)	72 (52.2)	42 (5.3)	15 (35.7)
Others: *Enterobacteriaceae* species	48 (3.8)	-	15 (2.0)	-
*Pseudomonas aeruginosa*/carbapenem	341 (15.4)	182 (53.3)	54 (7.9)	32 (59.3)
*Acinetobacter baumannii/*carbapenem	253 (11.4)	204 (80.6)	60 (8.7)	42 (70.0)
Others: non-fermenters	52 (2.3)	-	6 (0.8)	-
Total	1245 (56.1)	729 (58.0)	280 (35.6)	149 (53.2)
*Candida albicans*	107 (4.8)	-	32 (4.1)	-
Other *Candida* spp.	98 (4.4)	-	35 (4.4)	-
Other yeasts	19 (0.9)	-	7 (0.9)	-
Total	224 (10.1)	-	74 (9.4)	-

*Phenotypic resistance of the microorganisms.

In the 787 blood isolates, the most frequently observed groups/pathogens were CoNS (45.2%), followed by GNB (35.6%) and yeast (9.4%). *Enterobacteriaceae* and non-fermenters were observed in 57.1% (160/280) and 48.2% (120/280) of the isolates, respectively. Overall, the most prevalent bloodstream infection (BSI) agents were CoNS (45.2%), followed by *A. baumannii (*8.7%) and *P. aeruginosa* (7.9%).

Among the major phenotypes, the most common ones identified from all-site infections and BSIs were oxacillin-resistant CoNS, (89.9% and 87.4%, respectively), carbapenem-resistant *A. baumannii*, and wide-spectrum cephalosporin*-* resistant *Klebsiella* spp. (76.7% and 65.1%, 80.6% and 70.0%, respectively).

Brazilian studies have evaluated HCAI epidemiology in ICUs. The findings indicate a high prevalence in Brazil^8^, with clear evidence that the burden of these infections is higher in developing countries with limited resources^2^ ^,^ ^10^. In Brazil, both a higher prevalence of infection in ICUs (51.6%) and a clear association between infection and mortality (37.6%) are noted^11^. 

In the present study, GNB comprised most of the isolates, and *P. aeruginosa* and *A. baumannii* were the predominant organisms. However, BSIs are the most worrisome, and CoNS were the most common strain in these infections. GNB accounted for a significant percentage of these cases (35.6%), while *Enterobacteriaceae* family members (57.1%) and non-fermenters (42.0%) were among the most noteworthy pathogens identified in BSIs. 

The present study also demonstrated high incidence rates of infections and infection episodes. The overall mortality rate (within 30 days) among patients with infections was 38.0%, higher than the average hospital mortality rate (26.4%). The most common HCAI sites were the lungs (pneumonia) and the bloodstream; these accounted for one-third of all HCAIs and were associated with the use of invasive procedures^1^ ^,^ ^12^ ^,^ ^13^. These infections are a leading cause of death in countries with limited resources, and the mortality rates are higher than those in high-income countries^14^ ^,^ ^15^.

The increasing resistance to antimicrobial agents^16^ is a significant concern in low- and middle-income countries. It is amplified by several factors that include increasing antibiotic use and poor antibiotic control. Additionally, antimicrobial resistance is highly prevalent in hospitals and ICUs, as reported herein, which leads to limitations in infection prevention^7^.

In the present study, resistant/multidrug-resistant pathogens were the most frequently isolated pathogens from patients in the ICU and were present in most all-site and bloodstream isolates. Antimicrobial resistance is a growing challenge in the care of critically ill patients, particularly because it significantly increases morbidity, mortality rates, and costs related to ICU infections^17^ ^,^ ^18^. Oxacillin-resistant CoNS, carbapenem-resistant *A. baumannii,* and broad-spectrum cephalosporin-resistant *Klebsiella* spp. phenotypes were observed at high frequencies at all HCAI sites, mainly in BSIs.

This study has limitations due to its observational and retrospective nature and because it was performed at a single center. Our results may not be generalizable, and larger prospective studies are needed to confirm them.

In conclusion, ICU-acquired infections were common and often associated with the presence of resistant microorganisms. The most frequent ICU-acquired infections were BSIs in patients with positive blood cultures. Globally, the etiology of hospital infections includes mainly GNB as the most prevalent microorganisms, with a higher proportion of resistant organisms among key pathogen isolates. However, CoNS also frequently cause BSIs. This study provides information that will assist physicians in adopting more effective approaches for treating infections acquired in the hospital.
